# Changes in Proteinuria on the Risk of All-Cause Mortality in People with Diabetes or Prediabetes: A Prospective Cohort Study

**DOI:** 10.1155/2017/8368513

**Published:** 2017-09-27

**Authors:** Yang Sun, Anxin Wang, Xiaoxue Liu, Zhaoping Su, Junjuan Li, Yanxia Luo, Shuohua Chen, Jianli Wang, Xia Li, Zhan Zhao, Huiping Zhu, Shouling Wu, Xiuhua Guo

**Affiliations:** ^1^Department of Epidemiology and Health Statistics, School of Public Health, Capital Medical University, Beijing, China; ^2^Beijing Municipal Key Laboratory of Clinical Epidemiology, Capital Medical University, Beijing, China; ^3^Department of Neurology, Beijing Tiantan Hospital, Capital Medical University, Beijing, China; ^4^Department of Cardiology, Tangshan People's Hospital, North China University of Science and Technology, Tangshan, China; ^5^Department of Nephrology, Kailuan Hospital, North China University of Science and Technology, Tangshan, China; ^6^Department of Cardiology, Kailuan Hospital, North China University of Science and Technology, Tangshan, China; ^7^Department of Rehabilitation, Kailuan Hospital, North China University of Science and Technology, Tangshan, China; ^8^Department of Mathematics and Statistics, La Trobe University, Melbourne, Australia; ^9^State Key Laboratory of Transducer Technology, Institute of Electronics, Chinese Academy of Sciences, Beijing, China; ^10^University of Chinese Academy of Sciences, Beijing, China

## Abstract

**Background:**

Proteinuria has been related to all-cause mortality, showing regression or progression. However, few studies have focused on the relationship between proteinuria changes and all-cause mortality. The main purpose of this paper is to examine the associations between proteinuria changes and all-cause mortality in people with diabetes or prediabetes.

**Methods:**

Dipstick proteinuria at baseline and a 2-year follow-up were determined in the participants attending the Kailuan prospective cohort study. Participants were then divided into three categories: elevated proteinuria, stable proteinuria, and reduced proteinuria. Four Cox proportional hazard models were built to access the relations of proteinuria changes to all-cause mortality, adjusting for other confounding covariates.

**Results:**

A total of 17,878 participants were finally included in this study. There were 1193 deaths after a median follow-up of 6.69 years. After adjusting for major covariates and proteinuria at baseline, mortality risk was significantly associated with elevated proteinuria (hazard ratio (HR): 1.54, 95% confidence interval (CI): 1.33–1.79) and reduced proteinuria (HR: 0.70, 95% CI: 0.55–0.89), compared to those with stable proteinuria.

**Conclusion:**

Proteinuria changes were independently associated with mortality risk in either diabetic or prediabetic population.

## 1. Introduction

Proteinuria is an independent risk factor for mortality in both general- and high-risk populations, such as people with type 2 diabetes mellitus (T2DM) [1] and cardiovascular disease [2] and the elderly [3]. However, most studies have considered it at one point in time and did not assess the effect of changes in proteinuria on the future risk. Patients with diabetes are more likely to develop proteinuria, indicating an early glomerular injury [4], and are at high risk for renal failure [5], cardiovascular disease [6], and other adverse clinical outcomes, risks that are further increased at higher levels of proteinuria [7]. The impact of changes in proteinuria on mortality in patients with diabetes is needed to be explored.

Prediabetes, typically defined as blood glucose levels above normal but below diabetes thresholds, is a risk state for diabetes with an annualized conversion rate of 5%–10% [8]. The prevalence of prediabetes is increasing worldwide, and it is projected that over 472 million people will have prediabetes in 2030 according to the International Diabetes Federation [9]. The greatest absolute rises are expected in Southeast Asia, including China [9]. Several studies showed that insulin resistance might be one of the physiological links between prediabetes and renal dysfunction, as reflected by proteinuria [10, 11]. Recently, a meta-analysis reported that prediabetes was associated with increased all-cause and cardiovascular mortality [12]. Thus, it is important to estimate the association between changes in proteinuria and mortality in the prediabetic population.

Given the limited information on mortality outcomes associated with changes in proteinuria in people with diabetes or prediabetes, this study aimed to determine the associations between changes in proteinuria over a 2-year period and all-cause mortality in a large Chinese population from the Kailuan prospective study [13, 14].

## 2. Methods

### 2.1. The Kailuan Study

The Kailuan study is a prospective population-based cohort study of 101,510 people (81,110 males and 20,400 females, 18–98 years old) in the Kailuan community located in the Tangshan area of northern China [13]. The participants consisted of on-job and retired workers in the service of the Kailuan Coal Mine Group Corporation and residing in the Kailuan community. The design, methods, rationale, and examination details of the Kailuan cohort study were previously published elsewhere [15]. In brief, all participants accepted periodic health examinations, including questionnaire interviews, anthropometric measurements, clinical examinations, and laboratory assessments.

**Figure 1:**
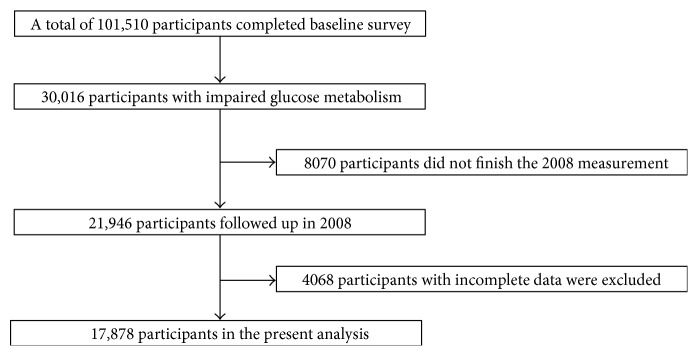
Flowchart of the study cohort.

The current study excluded the participants with normal glucose tolerance at baseline (*n* = 71,494), those who did not finish the second measurement (*n* = 8,070), or those with incomplete data (*n* = 4,068). Therefore, the remaining 17,878 participants were included in our analyses, which consist of 5590 diabetes and 12,288 prediabetes. The summary details of the inclusion and exclusion criteria for the study cohort have been included in Figure 1.

The study followed the guidelines of the Helsinki Declaration and was approved by the Ethics Committees of both the Kailuan Hospital and Beijing Tiantan Hospital. All participants provided their written informed consent.

### 2.2. Definition of Changes in Proteinuria

Morning urine samples were collected at both baseline (2006) and the 2-year follow-up (2008). Women were asked to collect urine samples during their nonmenstrual period. A freshly obtained urine sample was first visually examined for 1 minute by a trained physician (H12-MA and DIRUI N-600). Using a color scale, the results of the urine strip test were semiquantified as five degrees (none, trace, 1+, 2+, and 3+). The changes in proteinuria were defined as proteinuria degree at a 2-year follow-up minus proteinuria degree at baseline. Participants were classified as elevated proteinuria (difference value more than 0), stable proteinuria (difference value equals 0), and reduced proteinuria (difference value less than 0). “Per degree decrease of proteinuria changes” was defined as one proteinuria degree decline from the baseline (2006) to the 2-year follow-up (2008).

### 2.3. Follow-Up and Outcome Ascertainment

Participants were followed via face-to-face interviews at every 2-year routine medical examination until December 31, 2015, or until the time of death. Participants underwent a clinic examination, and any fatal events were collected through review of death certificates from the provincial vital statistics offices, hospital records, medical insurance data, and interviews with next of kin, relatives, or eyewitnesses, where such undertakings were possible. Any participants still alive at the end of the study will be recorded as censored.

### 2.4. Assessment of the Main Measurements and Other Potential Covariates

The candidate baseline variables presented in Table 1 were chosen for their common availability and use in previous studies [16]. The demographic data and information about lifestyle characteristics and history of diseases were obtained using questionnaires that were administered by trained doctors of the hospitals.

The classification of each category variable has been described elsewhere in some detail [17]. To further clarify, the physical activity group “active” was defined as had ≥80 minutes of activity per week and “inactive” group had <80 minutes of activity per week. Body mass index (BMI) was calculated according to the equation BMI = weight (kg)/height (m)^2^. Blood samples were collected in the morning after an overnight fast in the 11 hospitals and analyzed at the central laboratory of the Kailuan Hospital. Fasting plasma glucose (FPG) was measured using the hexokinase/glucose-6-phosphate dehydrogenase method. Triglyceride (TG), total cholesterol (TC), high-density lipoprotein (HDL), and low-density lipoprotein (LDL) levels were all measured enzymatically. Estimated glomerular filtration rate (eGFR) at baseline in 2006 and a follow-up in 2008 were calculated using the Chronic Kidney Disease Epidemiology Collaboration equation [18].

As we clarified before [14], hypertension was defined as systolic blood pressure ≥ 140 mmHg or diastolic blood pressure ≥ 90 mmHg, any use of antihypertensive drugs, or self-reported history of hypertension. Diabetes was defined as fasting glucose level ≥ 7.0 mmol/l, any use of glucose-lowering drugs, or any self-reported history of diabetes. Prediabetes was defined as a fasting glucose level between 5.6 mmol/l and 6.9 mmol/l [19] recognized by the American Diabetes Association. Dyslipidemia was defined as serum TG ≥ 1.69 mmol/l, LDL ≥ 3.62 mmol/l, HDL ≤ 1.04 mmol/l, any use of lipid-lowering drugs, or any self-reported history of dyslipidemia.

### 2.5. Statistical Analyses

SAS version 9.4 (SAS Institute, Cary, NC, USA) was used for our statistical analyses. The differences in continuous and categorical variables across the 3 groups for changes in proteinuria were assessed using ANOVA (Kruskal-Wallis) or *χ*^2^ tests, as appropriate. Four multivariable Cox proportional hazard models were built to adjust for different confounding covariates: (1) Model 1: adjusted for the levels of baseline proteinuria; (2) model 2: adjusted for age, gender, and baseline proteinuria; (3) model 3: adjusted for age, gender, level of education, income, smoking, alcohol abuse, amount of physical activity, BMI, and baseline proteinuria; (4) model 4: adjusted for variables in model 3 plus history of hypertension, diabetes, dyslipidemia, TC, TG, LDL, HDL, FPG, and creatinine. Furthermore, subgroup analyses were carried out on diabetes and prediabetes groups separately. Sensitivity analyses were also performed to verify the robustness of the study finding after excluding the population with eGFR less than 30 ml/min/1.73 m^2^ [20], which is to eliminate the possibility that impaired renal function may have an effect on the relationship between proteinuria changes and mortality. All reported *P* values were based on 2-sided tests of significance, and *P* < 0.05 was deemed statistically significant.

## 3. Results

### 3.1. Baseline Data

Baseline characteristics of the recruited participants stratified by changes in proteinuria are presented in Table 1. Of the 17,878 eligible participants, 14,032 participants (78.49%) had stable proteinuria, while 2299 (12.86%) had elevated proteinuria (proteinuria in 2008—proteinuria at baseline > 0) and 1547 (8.65%) had reduced proteinuria (proteinuria in 2008—proteinuria at baseline < 0). Participants who experienced a certain change in proteinuria (in either direction) were more likely to be older, had higher BMI, FPG, TC, TG, LDL, HDL, and creatinine, and were more frequent to have comorbidities, in comparison to those with stable proteinuria (all *P* < 0.05).

There were a total of 1193 deaths during a median follow-up of 6.69 years. The mortality rate is 6.67%. Among the three groups, the elevated proteinuria group had the highest death rate (11.61%) and the stable proteinuria group had the lowest death rate (5.59%). The difference was statistically significant (*P* < 0.0001).

### 3.2. Changes in Proteinuria and Risk of All-Cause Mortality

The hazard ratios (HRs) for the risk of death are shown in Table 2. Compared to those with stable proteinuria, participants with elevated proteinuria had an unadjusted HR of 1.92 (95% confidence interval (CI) = 1.66–2.23). Even after adjusting for confounders, the relationships still remained significant (model 2–4: HR = 1.67, 95% CI = 1.44–1.94; HR = 1.66, 95% CI = 1.43–1.92; and HR = 1.54, 95% CI = 1.33–1.79, resp.). While the reduced proteinuria group had an unadjusted HR of 0.75 (95% CI = 0.60–0.95) compared to the stable proteinuria group and even after adjusting for confounders, the relationships also remained significant (model 2–4: HR = 0.71, 95% CI = 0.56–0.90; HR = 0.71, 95% CI = 0.56–0.90; and HR = 0.70, 95% CI = 0.55–0.89, resp.). Therefore, we can see from model 4 that elevated proteinuria over a two-year period was associated with a 54% increase in the odds of all-cause mortality, and reduced proteinuria was associated with a 30% decrease in the odds of all-cause mortality compared to stable proteinuria.

In addition, four extra COX models were performed using the raw continuous proteinuria difference value to identify the relationship between per degree decrease of proteinuria changes and mortality risk. For per degree decrease of proteinuria changes, an unadjusted HR of 0.71 (95% CI = 0.67–0.75) was detected. After adjusting for confounders, per degree decrease in proteinuria changes could reduce the risk of death by 22 percent based on model 4.

To explore whether the hazard ratios are different between people with diabetes or prediabetes, the interaction term between changes in proteinuria and diabetes status was then taken into consideration based on model 4. The results showed that the interaction was not significant (*P* = 0.4274), indicating that prediabetic people are in the same risk of death with diabetic people when presenting elevated proteinuria.

### 3.3. Sensitivity Analysis

The results appeared to be more robust after excluding the population with eGFR less than 30 ml/min/1.73 m^2^. After adjusting for demography factors and laboratory indices, the associations between elevated proteinuria (HR = 1.55, 95% CI = 1.34–1.81) or reduced proteinuria (HR = 0.71, 95% CI = 0.56–0.90) and mortality risk were still found to be statistically significant in model 4 (Table 3). Per degree decrease of proteinuria changes contributed to the 22% decline of mortality risk. The interaction between changes in proteinuria and diabetes status was still not found (*P* = 0.3510).

## 4. Discussion

This study investigated the relationship between the proteinuria alterations, measured by repeated urine dipstick two years apart to future mortality risk among people with diabetes or prediabetes from a large longitudinal cohort. The results showed that elevated proteinuria was an independent risk factor for all-cause mortality while reduced proteinuria was a protective factor for all-cause mortality in people with diabetes or prediabetes. Per degree decrease of proteinuria changes contributed about 22% lower risk of mortality. The associations remained significant even after adjusting for confounding risk factors (age, gender, level of education, income, smoking, alcohol abuse, amount of physical activity, BMI, history of hypertension, diabetes, dyslipidemia, FPG, TC, TG, LDL, HDL, creatinine, and proteinuria at baseline).

There is growing awareness that chronic kidney disease is an important risk factor for mortality [21]. A number of cohort studies using dipstick proteinuria as an exposure have found evidence for an increase in total mortality [22, 23]. By contrast, the association between changes in proteinuria and mortality has rarely been studied. In 2014, Rein et al. reported that a rapid decline in kidney function is a powerful and independent new risk marker for death and vascular events [24]. In the study of Tan et al. in patients with T2DM, people with no remission of proteinuria had the worst prognosis of death, and failure to attain remission of proteinuria was associated with increased risk of mortality [25]. These findings indicate that a decline of the baseline proteinuria can be viewed as a meaningful change in kidney function, and further, consideration of change in kidney function adds prognostic information to improve mortality risk prediction beyond the patients' last proteinuria measurement [26]. The current study was in accordance with previous studies, by adding evidence that participants with elevated proteinuria are in high risk of mortality, even after adjusting for major covariates and baseline proteinuria.

The cut point of fasting glucose level for defining prediabetes was reduced from 6.1 mmol/l to 5.6 mmol/l, according to the 2003 ADA guideline [27]. Prospective studies had showed that the risk of cardiovascular disease and mortality was increased in people with fasting glucose level between 5.6 mmol/l and 6.9 mmol/l [28, 29]. The Asturias study reported that lowering the cutoff point optimized its ability to predict diabetes in a Spanish population, and the addition of other risk factors such as impaired glucose tolerance, hypertriglyceridemia, and overweight to IFG could stratify diabetes risk better [30]. There is evidence that the knowledge that one has impaired fasting glucose increased the motivation to exercise and diet, which resulted in a 58% reduction in the risk of progression to diabetes according to the Diabetes Prevention Program [31]. The present study used 5.6 mmol/l as the cut point of prediabetes. The interaction between changes in proteinuria and diabetes status was not found in our study, demonstrating the same risk of death when presenting elevated proteinuria in the two populations. It should serve as a reminder that people with prediabetes are also in high-risk groups for death if they had elevated proteinuria.

Therefore, annual screening of proteinuria is suggested for the diabetic and prediabetic population [32]. The standard urine dipstick test seems to be an ideal screening tool due to its ease of use and low cost. One potential implication of this study suggests that urine dipstick screening at a 2-year interval for detection of proteinuria changes may facilitate early identification of individuals at the highest risk for all-cause mortality, especially for those who are found to have elevated proteinuria during follow-up. Proteinuria may be a therapeutic target in addition to glucose management in specific population [33]. Thus, this early identification may allow early intervention and possibly improve outcomes.

The mechanisms why elevated proteinuria increases the risk of mortality remain uncertain. One of the principal kidney-specific mechanisms involved in the association has been proposed to be endothelial dysfunction. There is evidence that declining kidney function may directly aggravate oxidative stress, endothelial dysfunction, or activation of the renin-angiotensin system [34–36] which may lead to the increased concentration of asymmetric dimethylarginine inhibiting generation of nitric oxide and stimulating production of superoxide and cytokines [37]. In addition, worsening kidney function may result in decreased appetite, decreased physical function, and overall frailty [38, 39], thus indirectly contributing to a higher mortality risk.

Strengths of our study include the prospective design, use of a large cohort, long follow-up period, availability of repeated measurements of proteinuria, and use of proteinuria changes to estimate the risk of mortality. We also acknowledge several limitations of this analysis. First, one of the limitations is the unbalanced distribution of gender in the Kailuan cohort study considering most of the participants are male coal miners. Demographic characteristics and risk factors may differ; therefore, the findings may not be generalized directly to the general Chinese population. Second, this association with all-cause mortality for an overall cohort is unclear due to this observational study design. Although we carefully adjusted for potential risks factors and baseline proteinuria, the possibility of residual confounding still remained. Third, since proteinuria is determined by dipstick method, we never know if the qualified albuminuria is better to use to estimate the association. Fourth, this study lacks information about glycated hemoglobin due to the high cost for our large study samples, which is an important index for diagnosis of diabetes and prediabetes. Fifth, the disease duration and records of medications were not included in this study, which may have an effect on our results.

## 5. Conclusions

Proteinuria changes were independently associated with mortality risk in either diabetic or prediabetic population. These findings may help clinicians to interpret change in the outpatient setting and give more attention on prevention for people with prediabetes or diabetes. With the development of medicine, therapies targeted at proteinuria remission for all-cause mortality need further investigation.

## Figures and Tables

**Table 1:** Characteristics of study participants at baseline by changes in proteinuria.

Variable	Total	Changes in proteinuria			*P*
		Elevated proteinuria	Stable proteinuria	Reduced proteinuria	
Number of participants	17,878 (100.00)	2299 (12.86)	14,032 (78.49)	1547 (8.65)	
Age in years, mean (SD)	52.87 (10.93)	54.03 (11.41)	52.63 (10.76)	53.25 (11.53)	<0.0001
Gender female, *n* (%)	3027 (16.93)	326 (14.18)	2458 (17.52)	243 (15.71)	0.0002
High school or above, *n* (%)	3527 (19.73)	376 (16.35)	2878 (20.51)	273 (17.65)	<0.0001
Income ≥ 800RMB/month, *n* (%)	2751 (15.39)	302 (13.14)	2252 (16.05)	197 (12.73)	<0.0001
Current smoker, *n* (%)	6827 (38.19)	872 (37.93)	5372 (38.28)	583 (37.69)	0.8672
Current alcohol, *n* (%)	7613 (42.58)	899 (39.10)	6085 (43.37)	629 (40.66)	0.0002
Active physical activity, *n* (%)	3360 (18.79)	402 (17.49)	2640 (18.81)	318 (20.56)	0.0570
BMI, kg/m^2^, mean (SD)	25.78 (3.45)	26.23 (3.50)	25.83 (3.43)	26.15 (3.56)	<0.0001
Hypertension, *n* (%)	9235 (51.66)	1362 (59.24)	6893 (49.12)	980 (63.35)	<0.0001
Diabetes, *n* (%)	5590 (31.27)	945 (41.10)	4026 (28.69)	619 (40.01)	<0.0001
Prediabetes, *n* (%)	12,288 (68.73)	1354 (58.90)	10,006 (71.31)	928 (59.99)	
Dyslipidemia, *n* (%)	7935 (44.38)	1120 (48.72)	6055 (43.15)	760 (49.13)	<0.0001
Fasting plasma glucose, mmol/l, mean (SD)	7.04 (2.26)	7.55 (2.74)	6.90 (2.11)	7.53 (2.56)	<0.0001
Total cholesterol, mmol/l, mean (SD)	5.19 (1.11)	5.16 (1.20)	5.18 (1.08)	5.30 (1.23)	0.0061
Triglycerides, mmol/l, mean (SD)	1.95 (1.67)	2.12 (1.72)	1.90 (1.65)	2.10 (1.75)	<.0001
Low-density lipoprotein, mmol/l, mean (SD)	2.53 (0.93)	2.51 (1.10)	2.53 (0.90)	2.57 (1.01)	0.0433
High-density lipoprotein, mmol/l, mean (SD)	1.52 (0.40)	1.56 (0.42)	1.51 (0.39)	1.56 (0.44)	<0.0001
Creatinine, *μ*mol/l, mean (SD)	90.08 (32.09)	93.00(37.49)	89.89 (30.80)	87.44 (34.50)	<0.0001
All-cause mortality, *n* (%)	1193 (6.67)	267 (11.61)	784 (5.59)	142 (9.18)	<0.0001

SD: standard deviation; BMI: body mass index.

**Table 2:** Hazard ratios for the association between changes in proteinuria and all-cause mortality from 2006–2008.

	Changes in proteinuria			*P* trend	Per degree decrease
	Elevated proteinuria	Stable proteinuria	Reduced proteinuria		
All participants					
Model 1	1.92 (1.66–2.23)	1	0.75 (0.60–0.95)	<0.0001	0.71 (0.67–0.75)
Model 2	1.67 (1.44–1.94)	1	0.71 (0.56–0.90)	<0.0001	0.75 (0.71–0.79)
Model 3	1.66 (1.43–1.92)	1	0.71 (0.56–0.90)	<0.0001	0.75 (0.71–0.80)
Model 4	1.54 (1.33–1.79)	1	0.70 (0.55–0.89)	<0.0001	0.78 (0.73–0.82)
Diabetes^∗^					
Model 4	1.75 (1.43–2.15)	1	0.73 (0.53–0.99)	<0.0001	0.74 (0.69–0.80)
Prediabetes					
Model 4	1.34 (1.07–1.68)	1	0.68 (0.47–0.98)	0.0002	0.82 (0.75–0.90)

Model 1: adjusted for the levels of baseline proteinuria; model 2: adjusted for age, gender, and baseline proteinuria; model 3: adjusted for age, gender, level of education, income, smoking, alcohol abuse, amount of physical activity, body mass index, and baseline proteinuria; model 4: adjusted for variables in model 3 plus history of hypertension, diabetes, dyslipidemia, total cholesterol, triglycerides, low-density lipoprotein, high-density lipoprotein, fasting plasma glucose, and creatinine. ^∗^The *P* value of interaction between proteinuria changes and diabetes status for all-cause mortality is 0.4274.

**Table 3:** Sensitivity analysis by excluding the population with eGFR less than 30 ml/min/1.73 m^2^.

	Changes in proteinuria			*P* trend	Per degree decrease
	Elevated proteinuria	Stable proteinuria	Reduced proteinuria		
All participants					
Model 1	1.93 (1.66–2.23)	1	0.76 (0.60–0.97)	<0.0001	0.71 (0.67–0.76)
Model 2	1.68 (1.45–1.95)	1	0.72 (0.57–0.92)	<0.0001	0.75 (0.71–0.80)
Model 3	1.66 (1.43–1.93)	1	0.72 (0.57–0.92)	<0.0001	0.75 (0.71–0.80)
Model 4	1.55 (1.34–1.81)	1	0.71 (0.56–0.90)	<0.0001	0.78 (0.73–0.82)
Diabetes^∗^					
Model 4	1.76 (1.43–2.15)	1	0.73 (0.54–1.01)	<0.0001	0.74 (0.69–0.80)
Prediabetes					
Model 4	1.35 (1.07–1.69)	1	0.71 (0.49–1.02)	0.0005	0.82 (0.75–0.91)

Model 1: adjusted for the levels of baseline proteinuria; model 2: adjusted for age, gender, and baseline proteinuria; model 3: adjusted for age, gender, level of education, income, smoking, alcohol abuse, amount of physical activity, body mass index, and baseline proteinuria; model 4: adjusted for variables in model 3 plus history of hypertension, diabetes, dyslipidemia, total cholesterol, triglycerides, low-density lipoprotein, high-density lipoprotein, fasting plasma glucose, and creatinine. ^∗^The *P* value of interaction between proteinuria changes and diabetes for all-cause mortality is 0.3510.
